# LDL biochemical modifications: a link between atherosclerosis and aging

**DOI:** 10.3402/fnr.v59.29240

**Published:** 2015-12-03

**Authors:** Matilde Alique, Carlos Luna, Julia Carracedo, Rafael Ramírez

**Affiliations:** 1 Departamento Biología de Sistemas, Facultad de Medicina y Ciencias de la Salud, Universidad de Alcalá, Madrid, Spain; 2 Instituto Maimónides de Investigación Biomédica de Córdoba (IMIBIC), Hospital Universitario Reina Sofía, Universidad de Córdoba, Córdoba, Spain

**Keywords:** aging, atherosclerosis, endothelial damage, LDL modifications, oxidative stress

## Abstract

Atherosclerosis is an aging disease in which increasing age is a risk factor. Modified low-density lipoprotein (LDL) is a well-known risk marker for cardiovascular disease. High-plasma LDL concentrations and modifications, such as oxidation, glycosylation, carbamylation and glycoxidation, have been shown to be proatherogenic experimentally *in vitro* and *in vivo*. Atherosclerosis results from alterations to LDL in the arterial wall by reactive oxygen species (ROS). Evidence suggests that common risk factors for atherosclerosis raise the likelihood that free ROS are produced from endothelial cells and other cells. Furthermore, oxidative stress is an important factor in the induction of endothelial senescence. Thus, endothelial damage and cellular senescence are well-established markers for atherosclerosis. This review examines LDL modifications and discusses the mechanisms of the pathology of atherosclerosis due to aging, including endothelial damage and oxidative stress, and the link between aging and atherosclerosis.

Evidence from the past several decades has suggested that modification of low-density lipoprotein (LDL) – especially oxidative changes – mediates the pathogenesis of atherosclerosis in humans and animals (1, 2). Although aging is a major risk factor that precipitates atherosclerosis, there are many other factors that can cause the disease (3, 4). The major risk factors for atherosclerosis are serum lipid concentrations, smoking, and hypertension (5).

Gender appears to be another determinant (6). Men are approximately twice as likely to develop atherosclerosis compared with age-equivalent women. Yet, merely half of the variability in the incidence of atherosclerosis and coronary heart disease is because of these factors. Genetics might have some influence, but age-related conditions might have a more prominent function for the development of the disease (7). Furthermore, excess food intake affects obesity and diabetes, both of which are well-known independent risk factors of atherosclerosis and growing epidemics in an aging population (8).

The presence of multiple risk factors can accelerate the progression of atherosclerosis. Modified LDL has a significant function in the development of endothelial dysfunction (9), which is considered an early marker of atherosclerosis (10). More extensive modification of LDL induces oxidative stress and accelerates senescence in human endothelial progenitor cells and endothelial cells (11, 12). The hallmark of endothelial dysfunction is impaired endothelium-dependent vasodilatation, which is mediated by nitric oxide (NO). A defect in NO production or activity has been proposed as a significant mechanism of endothelial dysfunction and a contributor to atherosclerosis (10).

The focus of this study is to discuss the function of protein modifications, particularly those to LDL, in aging-induced atherosclerosis, and how these molecules mediate senescence-related signaling and endothelial damage during atherosclerosis.

## Chemical modifications to LDL related to atherosclerotic processes

Cardiovascular disease (CVD) remains the leading cause of mortality in developed countries, and LDL analysis is one of the most widely used diagnostic indices to evaluate and predict atherosclerosis risk (2). The levels of LDL and other lipoproteins, however, usually have limited predictive value because many factors affect LDL concentrations in the arterial wall, the rate and extent of LDL modifications, and the accumulation of LDL in vascular cells and disease progression (13).

Several chemically modified LDL species have been reported since the 1970s, including oxidized LDL (oxLDL) (14, 15), acetylated LDL (acLDL) (16, 17), ethylated (17), methylated (17), and glycated LDL (gLDL) (18). Based primarily on studies of oxLDL and acLDL, endothelial cell injury by modified LDLs has been commonly accepted to initiate atherosclerotic processes (19). These LDLs also promote vascular injury by increasing oxidative stress and accelerating senescence of endothelial progenitor cells through modifications and damage to DNA (12).

It is widely believed that the modification of various components of LDL alters the properties that contribute to its atherogenic effects when interacting with cells of the arterial wall. Specifically, changes in apoliprotein B (apoB; the surface protein of LDL) destroy the ability of LDL to bind LDL receptor (20). LDL modifications and especially oxidation might mediate the induction of atherogenesis through scavenger receptors (SRs) on macrophages and endothelial cells (21).

**Fig. 1:**
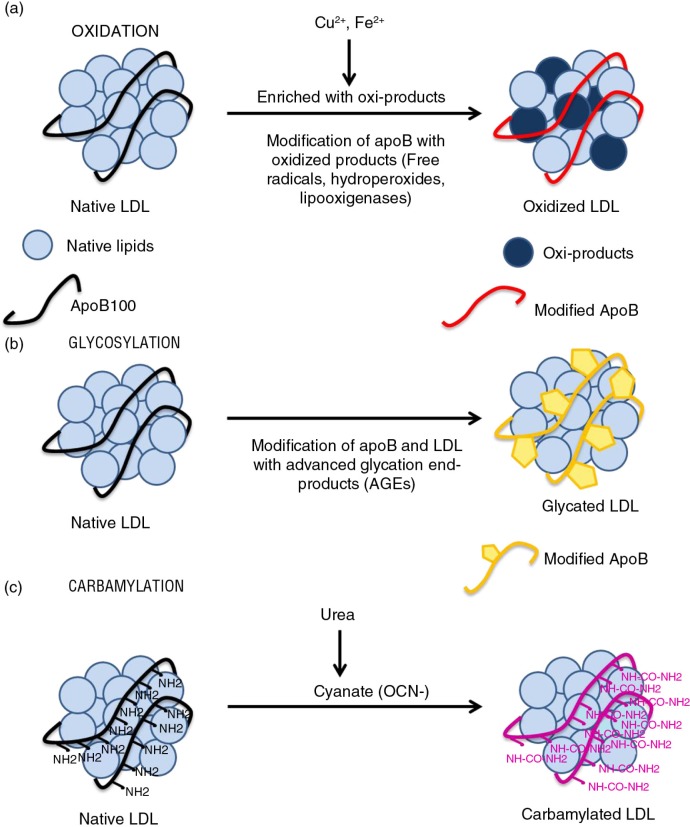
LDL modifications. (a) Oxidation: oxidized product-induced native LDL oxidation and modifications of apoB amino acids. (b) Glycosylation: modification of LDL and apoB by advanced glycosylation end-products (AGEs). (c) Carbamylation: cyanate from urea, which binds to NH2 groups in proteins – inducing their carbamylation – is generated by spontaneous dissociation from urea.

Several pathways and processes lead to harmful modification of LDL (Fig. 1).

### LDL oxidation

In 1981, Henriksen et al. (22) discovered that native LDL, which is incubated overnight with cultured endothelial cells, converts to a form (endothelial cell-modified LDL) that is recognized specifically by peritoneal macrophages with high affinity. They proposed that this endothelium-induced modification is the step that permits rapid LDL uptake and foam cell formation. Later studies reported that during its incubation with endothelial cells (and with several cell types), LDL undergoes oxidative changes (23), constituting the basis of the oxidative modification hypothesis of atherogenesis.

Oxidative modification of lipid and proteins occurs frequently in many pathophysiological processes *in vivo*, and it is well established that LDL undergoes oxidative alterations that confer atherogenic properties to it (24).

Under oxidative stress (free radicals, hydroperoxides, and lipooxigenases), lipid molecules that contain native lipids in LDL are easily oxidized. A variety of lipid oxidation products is formed, and subsequently, apoB is covalently modified by these oxidized lipids (25) (Fig. 1a).

OxLDL is taken up by SRs on macrophages, which then become lipid-laden foam cells, the pathological hallmark of early atherosclerotic lesions (26). The concentration of LDL that is needed to induce foam cell formation (2 mg/ml) is 40-fold greater than that of oxLDL (50 μg/ml) (27).

OxLDL has a wide range of properties that are expected to be proatherogenic, many of which are affected by oxidized phospholipids in oxLDL (28). Oxidized phospholipid products are also the principal epitopes that are recognized by autoantibodies as oxLDL and the major structural feature by which SRs recognize oxLDL as a ligand.

Recently, oxLDL has been associated with changes in endothelial cell homeostasis through the suppression of important endothelial microRNAs (miRNAs) (29). Further, the miRNAs are a link between endothelial injury and inflammation and can mediate inflammatory activation and lipid accumulation in macrophages during atherosclerosis (29).

### LDL glycosylation

Clinical studies have demonstrated increased levels of advanced glycosylation end products (AGEs) on LDL from diabetics compared to normal individuals (30). AGEs accumulate continuously on long-lived vessel wall proteins with aging and at higher rates in diabetes (31). One of the mechanisms of accelerated atherosclerosis in diabetes is the non-enzymatic reaction between glucose and proteins or lipoproteins in arterial walls. The degree of non-enzymatic glycation is determined primarily by glucose concentrations and the time of exposure (31). Although non-enzymatic glycosylation of LDL occurs in all subjects, it has more adverse effects in people with diabetes mellitus.

Glycosylation of LDL apoB (Fig. 1b) occurs mainly on positively charged lysine residues in the putative LDL receptor-binding domain, which is essential for the specific recognition of LDL by LDL receptor (32). This modification leads to a loss of electropositive charges on gLDL, decreasing its affinity toward LDL receptor and consequently increasing its mean lifetime in plasma (33). Greater LDL glycosylation correlates with glucose levels, and AGE–apoB levels are up to fourfold higher in diabetic patients (30). Once formed, AGE-protein adducts are stable and virtually irreversible.

Glycosylation of apoB results in significant impairing of LDL receptor-mediated uptake, decreasing the *in vivo* clearance of LDL compared with native LDL (34). Thus, gLDL is poorly recognized by LDL receptor and binds preferentially to SRs on human macrophages. As LDL glycosylation enhances its uptake by human aortic intimal cells (30) and monocyte-derived macrophages (35) on stimulation of foam cell formation, the recognition of gLDL by the SR pathway is believed to promote intracellular accumulation of cholesteryl esters and atherosclerosis.

Prolonged hyperglycemia is now recognized to be the primary casual factor in the pathogenesis of diabetic complications (36, 37). Hyperglycemia induces a large number of alterations in vascular tissue that potentially accelerate atherosclerosis. Two major mechanisms have emerged that account for most of the pathological alterations observed in the vasculature of diabetic animals and humans: [1] non-enzymatic glycosylation of proteins and lipids and [2] oxidative stress. Notably, these mechanisms are not independent (38).

**Fig. 2:**
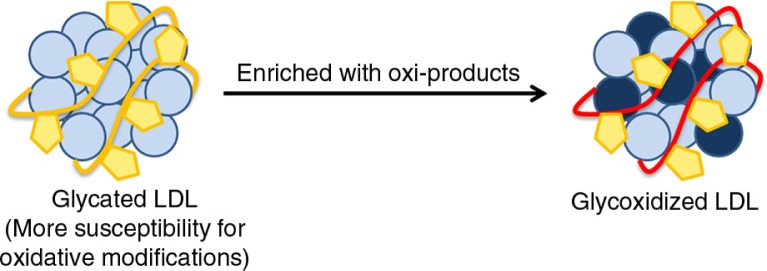
LDL double modification. The glycation of LDL particles renders it more prone to oxidation.

Another atherogenic effect of glycation is the increased susceptibility of gLDL to oxidative modification (39) (Fig. 2). Glycosylation process occurs on the apoB (40) and phospholipid (30) components of LDL. Therefore, glycation raises the susceptibility of LDL to oxidative modification (41), which is considered a critical step in its atherogenicity. Doubly modified LDL might have greater pro-atherosclerotic potential compared with gLDL. A number of reports from different groups have described shortened oxidative lag periods during Cu^2+^-mediated oxidation of gLDL and LDL in diabetic patients (42, 43). Thus, glycosylation of LDL is not only noxious per se but also it promotes oxidation of LDL.

### LDL carbamylation

Carbamylation is a post-translational and non-enzymatic modification in which amine-containing residues react with cyanate, a compound that derives from urea or thiocyanate. The carbamylation of LDL occurs due to spontaneous, non-enzymatic chemical modification of apoB by urea-derived cyanate. Urea dissociates spontaneously to cyanate and ammonia in aqueous solutions, elevating cyanate levels (OCN-) (44) (Fig. 1c). The active form of cyanate, isocyanic acid, reacts irreversibly with the NH2 and N-terminal groups of amino acids (45). When a molecule of cyanate is removed by carbamylation, a new molecule of cyanate is formed to restore the equilibrium between urea and cyanate. Carbamylation of a protein is usually associated with a partial loss of function (46, 47), but carbamylated proteins usually have no positive effect in humans or animals and are dispensable for normal metabolism.

Protein carbamylation is frequent in patients with chronic renal failure (CRF) and heavy smokers (48). CVD rates are up to 30 times higher in patients with CRF compared with the general population, and morbidity and mortality rates rise, even in the initial stages of the disease (48, 49). In particular, the incidence of atherosclerosis is high in CRF patients with uremia, but the pathogenic events that contribute to uremic atherosclerosis are poorly understood (50). As suggested above, modified LDLs are significant atherogenic factors (51, 52), and carbamylated LDL (cLDL) has been recognized to be a type of modified LDL (12). Essentially, carbamylation of LDL could be is an important mechanism that impacts high-risk atherosclerotic individuals with increased urea (renal insufficiency) or thiocyanate (tobacco smoking). LDL carbamylation is more extensive in patients with end-stage kidney disease, especially those with atherosclerosis (45, 53).

It remains unknown whether protein carbamylation induces atherosclerosis and whether the atherogenic process proceeds through LDL or other targets. Notwithstanding, there is a report that demonstrates the role of carbamylated plasma proteins have been implicated in the development of cardiovascular complications (54). For animals, uremic mice with high-plasma cLDL have more severe atherosclerosis (3). However, future therapies might be aimed at reducing cLDL and its effects.

## Endothelial damage and atherosclerosis

Endothelial dysfunction of large- and medium-sized arteries is characterized by impaired NO-mediated vasodilatation and appears to play a pivotal role in atherosclerosis; both endothelial dysfunction and atherosclerosis are induced by coronary artery disease risk factors, such as cigarette smoking, hypertension, diabetes mellitus, hyperhomocysteinemia, serum lipid concentrations, and hypercholesterolemia (55). Several processes, such as senescence and increased oxidative stress, affect endothelial dysfunction and the development of atherogenesis (56, 57).

### Aging endothelial cells

Aged endothelial cells become flatter and more enlarged and have an increasingly polypoid nucleus – all of which are associated with cellular senescence (58, 59). These changes are accompanied by alterations in cytoskeleton integrity, proliferation, angiogenesis, and cell migration. Senescent endothelial cells produce less NO (60) and release more endothelin-1 (ET-1) (61). Late-passage endothelial cells also downregulate adhesion molecules, vascular cell adhesion protein 1 (VCAM-1), and intracellular adhesion molecule-1 (ICAM-1); show increased activation of nuclear factor (NF)-κB; and experience greater susceptibility to apoptosis (59). In addition, NF-κB activation enhances endothelial cell senescence and can, therefore, reduce endothelial regeneration at sites that are prone to atherosclerosis (62).

**Fig. 3:**
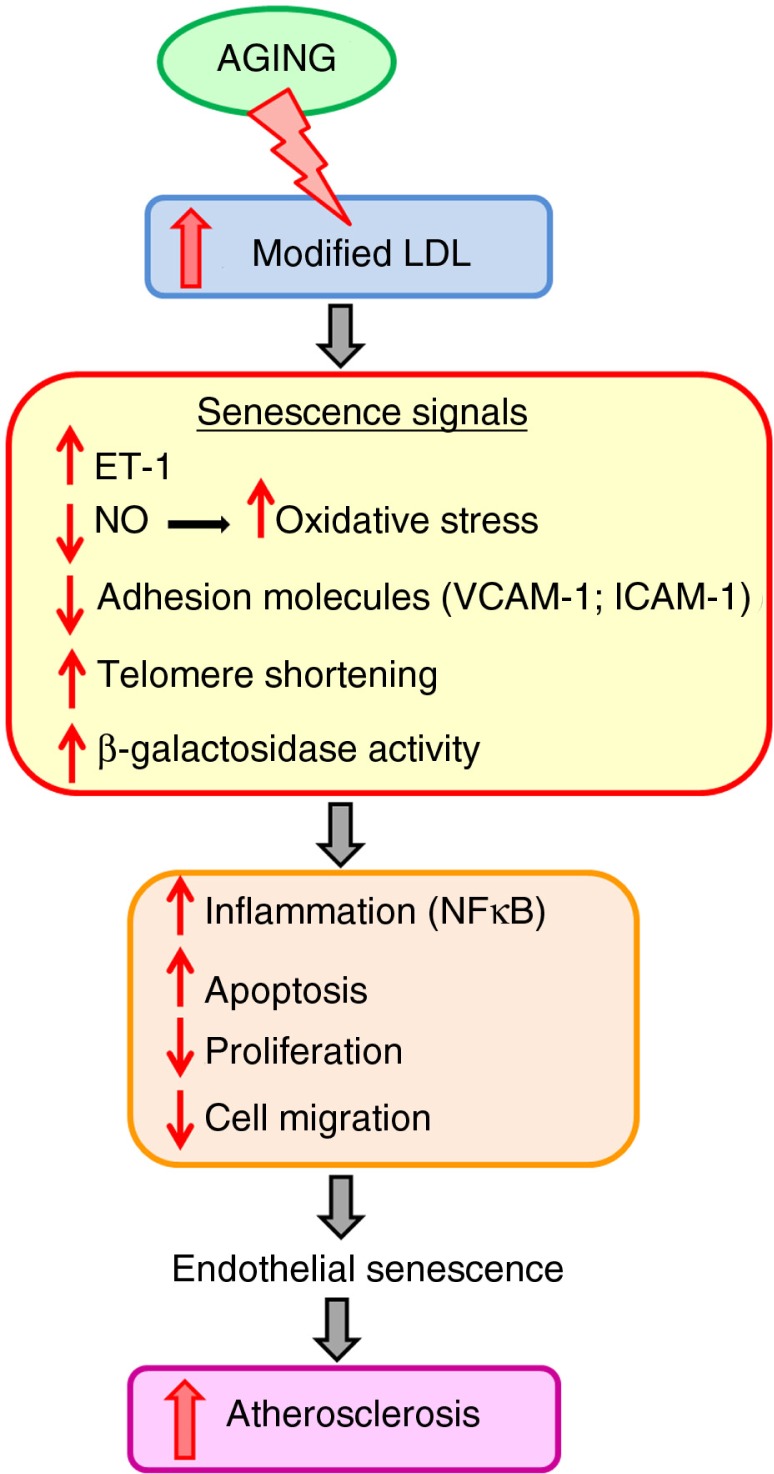
Mechanisms of endothelial cell senescence during aging, initiating the atherogenic process.

Furthermore, there are marked age-associated changes in function and activity (63). Thus, endothelial cell senescence is associated with a loss of endothelial cell function and a shift toward a pro-inflammatory and pro-apoptotic state – which are predicted to enhance monocyte migration into the vessel wall (64). In addition, it has been shown that both oxLDL and LDL induce accelerated senescence, as evidenced by telomere shortening and β-galactosidase activity (12). All of these processes correlate with increasing severity of atherosclerosis (Fig. 3).

### Oxidative stress in endothelial cells

NO is a free radical signaling molecule that is produced by nitric oxide synthase enzymes (65, 66). Some of the beneficial properties of NO include vasodilatation, promotion of endothelial cell survival, and inhibition of cell proliferation and migration (67), which might protect against atherosclerosis. Several studies have reported a decrease in nitric oxide activity with aging.

Oxidative stress might be involved more broadly in atherogenesis. Reactive oxygen species (ROS) are produced in the endothelium, smooth muscle cells, and adventitia. There is a significant amount of evidence that implicates them in vasomotor activity, smooth muscle cell growth, expression of adhesion molecules, apoptosis, activation of metalloproteinases and, of course, lipid oxidation (68). These oxidation-related processes can accompany oxidation of LDL or occur independently of it. Indeed, lipid peroxidation seems to be an important mechanism in the development of endothelial dysfunction in certain pathologies (69, 70).

## Aging and atherosclerosis

Age is a non-modifiable risk factor of atherosclerosis. Aging is not simply wear and tear but is an active process, like atherosclerosis, with which it shares mechanisms, such as endothelial dysfunction. Several studies have shown that endothelial cell function is compromised with aging and that endothelial cell senescence mediates the evolution of age-associated CVDs, such as atherosclerosis (71, 72). Older primates and rodents develop more extensive atherosclerosis than younger animals when both groups are fed an atherogenic diet (73).

Age-accelerated vascular injury is commonly considered to result from increased oxidative stress, leading to inflammation and endothelial dysfunction, but no definite mechanisms have been identified (74). The accumulation of oxidative damage is believed to contribute to aging and its associated diseases (75). Tissues from aged animals show an increased generation of ROS, leading to altered mitochondrial function, damage to vascular cells with age-associated remodeling, and oxidation of lipids, rendering them more atherogenic (76).

Age and other atherosclerotic risk factors upregulate pathways that increase ROS production, whereas antioxidant mechanisms are enhanced and decrease in aging (77, 78). Caloric restriction attenuates the increases in inflammation, oxidative stress, and endothelial dysfunction that accompany aging and extend the median lifespan in several animals (79, 80). Conversely, caloric excess that leads to obesity worsens these factors and promotes insulin resistance, metabolic syndrome, and diabetes, all of which are frequently associated with aging and are established contributors to the accelerated atherosclerosis (81, 82). Hypertensive patients are more prone to atherosclerotic lesions and acute ischemic events than normotensive individuals (83).

The risk factors of atherosclerosis are well known, including hypertension (84, 85), diabetes (86, 87), serum total LDL cholesterol (88–90), smoking (91, 92), and obesity (93, 94). Increasing evidence indicates that aging is also an important risk factor for atherosclerosis and persists as an independent contributor when all other factors are controlled for. Premature or accelerated vascular aging can be promoted by cardiovascular risk factors (95, 96), and cellular senescence is observed in patients with atherosclerosis. Atherosclerosis is thus a disease of organismal aging and cellular senescence.

Atherosclerosis is a condition caused by lipid-induced inflammation of the vessel wall that is orchestrated by the complex interplay of various types of cells, such as endothelial cells, smooth muscle cells, and macrophages. Hypercholesterolemia, especially high concentrations of serum LDL cholesterol – is considered a major factor of atherosclerosis. However, oxidation of LDL appears to have significant function in the early development of atherosclerosis through the formation of macrophage-derived foam cells on the arterial wall (97, 98). Macrophages bind and take up oxLDL particles – but not nonoxidized, native LDL particles – via SRs (99).

Aged vessels undergo several characteristic pathological processes, many of which are also seen in atherosclerosis (100, 101).

## Conclusions

CVD remains one of the most significant chronic diseases worldwide with regard to morbidity and mortality. Although CVD is associated with aging, accumulating evidence supports that CVD is linked to vascular cell senescence; considering that CVD occurs in aged people when vascular cells undergo replicative senescence and in patients whose risk factors promote premature senescence. In particular, senescence in endothelial cells seems to be the initial step in the cascade of events that lead to CVD. Thus, the factors that affect senescence in endothelial cells and the mechanisms by which they do so must be identified to develop new biomarkers and therapeutic tools against CVD. In this regard, determining how endothelial cells are affected by structural changes in lipoproteins, such as LDL, is a promising approach for future studies on CVD.
